# BAM15 improves oocyte quality against obesity via PPARγ-dependent mitochondrial function

**DOI:** 10.1093/procel/pwag035

**Published:** 2026-05-24

**Authors:** Rui-Jie Ma, Hao-Lin Zhang, Xuan Wu, Ping-Shuang Lu, Kun-Huan Zhang, Yu-Xuan Hou, Zi-Jian Wu, Yue Wang, Shao-Chen Sun

**Affiliations:** College of Animal Science and Technology, Nanjing Agricultural University, Nanjing 210095, China; College of Animal Science and Technology, Nanjing Agricultural University, Nanjing 210095, China; College of Animal Science and Technology, Nanjing Agricultural University, Nanjing 210095, China; College of Animal Science and Technology, Nanjing Agricultural University, Nanjing 210095, China; College of Animal Science and Technology, Nanjing Agricultural University, Nanjing 210095, China; College of Animal Science and Technology, Nanjing Agricultural University, Nanjing 210095, China; College of Animal Science and Technology, Nanjing Agricultural University, Nanjing 210095, China; College of Animal Science and Technology, Nanjing Agricultural University, Nanjing 210095, China; College of Animal Science and Technology, Nanjing Agricultural University, Nanjing 210095, China; Key Laboratory of Research on Clinical Molecular Diagnosis for High Incidence Diseases in Western Guangxi of Guangxi Higher Education Institutions, Affiliated Hospital of Youjiang Medical University for Nationalities, Baise 533000, China

**Dear Editor,** 

With the increasing prevalence of obesity, this chronic condition has become a global health issue. It raises risks of cardiovascular diseases and diabetes (Powell-Wiley et al., 2021) and impairs female reproduction by disrupting folliculogenesis and oocyte maturation. Mitochondrial dysfunction is recognized as the core mechanism underlying obesity-induced oocyte quality impairment. In obese oocytes, abnormal mitochondrial distribution and decreased membrane potential further lead to massive accumulation of reactive oxygen species (ROS) (Hou et al., 2016). Several interventions for oocyte damage caused by obesity have been reported. Metformin indirectly protects oocytes in obese rats by improving ovarian metabolism and ovulation (Zhang et al., 2024), while NMN partially rescues oocyte quality via modulating mitochondrial function and actin dynamics to alleviate cellular damage (Wang et al., 2022). Despite their protective effects, these agents still have limitations including unstable efficacy and unclear long-term safety. Based on this essential pathological mechanism, targeted mitochondrial intervention is a rational and promising therapeutic strategy.

BAM15 is a novel mitochondrial uncoupler. It promotes proton reflux independent of ATP synthase, enhancing electron transport chain efficiency and reducing ROS production (Kenwood et al., 2014). It counteracts diet-induced obesity and insulin resistance by elevating energy expenditure without altering feeding behavior (Axelrod et al., 2020), while also attenuating mitochondrial oxidative stress, inflammatory damage and splenic apoptosis to reduce mortality in sepsis (Tsuji et al., 2023). However, the effects and molecular mechanisms of BAM15 on the reproductive system, especially in oocytes, remain unclear. Therefore, this study aims to investigate whether BAM15 can reverse obesity-induced oocyte damage by targeted regulation of mitochondrial function and to clarify its molecular mechanisms.

In this study, we established a diet-induced obesity mouse model. Six-week-old female ICR mice were fed a standard chow or high-fat diet (HFD) for 12 weeks (Fig. 1A). HFD feeding significantly increased body weight, reduced ovulation rate and ovulated oocyte count (Fig. S1A and S1B). We then performed *in vitro* BAM15 treatment to rescue obese oocyte quality (Fig. 1A). Screening results showed that BAM15 had no significant effect on germinal vesicle breakdown (GVBD) in obese mouse oocytes at various concentrations (Fig. S1C), while 0.5 μmol/L BAM15 significantly elevated their first polar body (PB1) extrusion rate (Fig. 1B), which was selected for further research (named obese + BAM15 group). BAM15 treatment ameliorated fertilization defects in obese oocytes (Fig. S1D), with no significant difference in fertilized MII oocyte fragmentation rate detected among the control, obese and obese + BAM15 groups (Fig. S1E). Oocytes at the MII stage from obese mice showed disordered distribution of cortical granules, which could be restored by BAM15 supplementation (Fig. S1F). Moreover, BAM15 showed no effect on 2-cell embryo formation rate (Figs. 1A and S1G), but effectively alleviated obesity-induced blastocyst developmental impairment (Fig. 1A and 1C). These results indicated that BAM15 reversed obesity-associated oocyte quality and embryo developmental competence defects.

**Figure 1.**
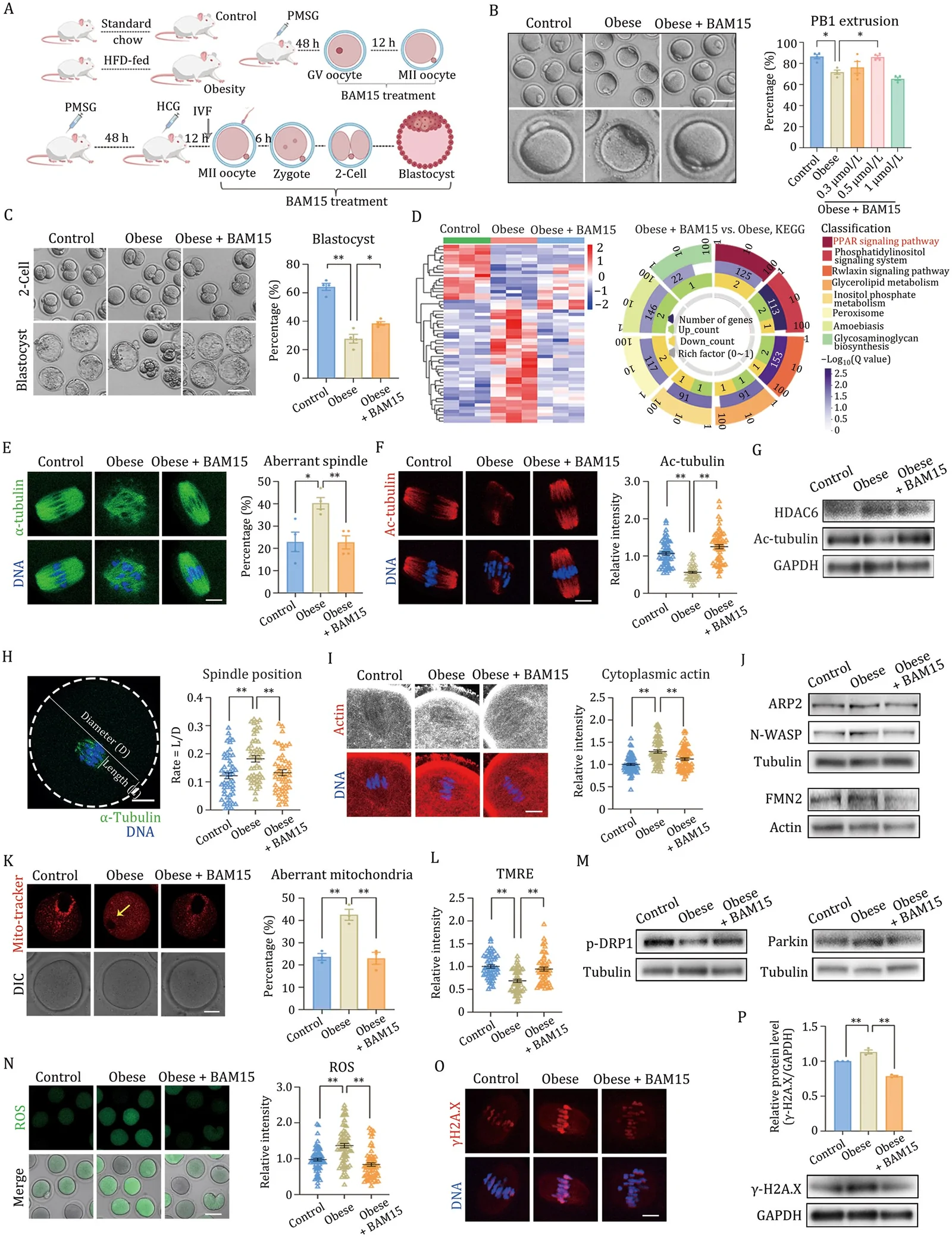
**BAM15 ameliorates developmental defects and improves oocyte quality in obese mice**. (A) Flowchart of HFD-induced obesity model establishment, and timeline chart of BAM15 supplementation during *in vitro* oocyte maturation and early embryonic development. IVF, *in vitro* fertilization. (B) Representative PB1 extrusion images after 12 h culture and quantitative rates from control (*n *= 127), obese (*n *= 145), and obese oocytes treated with 0.3, 0.5, and 1.0 μmol/L BAM15 (*n *= 130, 130, and 132) during *in vitro* maturation (IVM). Bar = 80 μm. (C) Representative blastocyst images and quantified formation rates at 120 h after IVF are shown for the control (*n *= 175), obese (*n *= 126), and obese + BAM15 (*n *= 144) groups. Blastocyst formation rates were calculated based on fertilized zygotes. Bar = 80 μm. (D) Heatmap showing hierarchical clustering of common differentially expressed genes (DEGs) in MI stage oocytes among control, obese, and obese + BAM15 groups. KEGG enrichment analysis of DEGs between obese + BAM15 and obese oocytes. (E) Representative images and abnormal spindle in oocytes from control (*n *= 68), obese (*n *= 67), and obese + BAM15 (*n *= 70) groups. Bar = 10 μm. (F) Representative images and relative fluorescence intensity of ac-tubulin in oocytes from control (*n *= 61), obese (*n *= 58), and obese + BAM15 (*n *= 62) groups. Bar = 10 μm. (G) Representative western blot bands of HDAC6 and ac-tubulin in MI stage oocytes from the control, obese, and obese + BAM15 groups. (H) The MI stage oocyte diameter (D) and spindle-to-cortex distance (L) were measured, and the L/D ratio was calculated to evaluate spindle migration toward the cell cortex. Statistical analysis was performed on the L/D ratio of oocytes from the control (*n *= 51), obese (*n *= 49), and obese + BAM15 (*n *= 50) groups. Bar = 20 μm. (I) Representative images and relative fluorescence intensity of cytoplasmic actin filaments in oocytes from control (*n *= 66), obese (*n *= 64), and obese + BAM15 (*n *= 72) groups. Bar = 10 μm. (J) Representative western blot bands of ARP2, N-WASP and FMN2 in MI stage oocytes from the control, obese, and obese + BAM15 groups. (K) Representative images and percentage of abnormal mitochondria (indicated by arrows) in oocytes from control (*n *= 55), obese (*n *= 49) and obese + BAM15 (*n *= 52) groups. Bar = 20 μm. (L) Relative fluorescence intensity of TMRE in MI oocytes from control (*n *= 52), obese (*n *= 51), and obese + BAM15 (*n *= 54) groups. (M) Representative western blot bands of pS616-DRP1 and Parkin in MI stage oocytes from the control, obese, and obese + BAM15 groups. (N) Representative images and relative fluorescence intensity of ROS in oocytes from control (*n *= 68), obese (*n *= 74), and obese + BAM15 (*n *= 65) groups. Bar = 80 μm. (O) Representative images of γ-H2A.X in oocytes from control, obese, and obese + BAM15 groups. Bar = 10 μm. (P) Representative western blot images and band intensity analysis of γ-H2A.X in MI stage oocytes of control, obese, and obese + BAM15 groups. Data are presented as mean ± SEM of three biological repeats. *, *P *< 0.05. **, *P *< 0.01.

Single-cell RNA sequencing was conducted to explore the underlying mechanism. PCA analysis verified well-clustered samples without outliers (Fig. S2A). Compared with control group, obese group exhibited obvious differentially expressed genes (DEGs) changes, which were reversed by BAM15 intervention, and 55 common DEGs were identified across three groups (Figs. 1D and S2B). The reliability of sequencing results was verified by qPCR (Fig. S2C and S2D; Table S1). Gene ontology (GO) analysis showed DEGs enriched in cytoskeleton, organelle components and energy-related functions, while Kyoto encyclopedia of genes and genomes (KEGG) revealed metabolic disorders and insulin resistance in obese oocytes, with the PPAR pathway as a key BAM15-mediated metabolic regulator (Figs. 1D, S2E and S2F). Based on GO enrichment, microtubule function assays showed spindle abnormalities, impaired microtubule assembly and abnormal microtubule acetylation in obese oocytes, all of which were ameliorated by BAM15 (Figs. 1E, 1F and S3A). STRING analysis confirmed a strong correlation between microtubule-related DEGs and HDAC6 (Fig. S3B). HDAC6 was upregulated and acetylated tubulin was decreased in obese oocytes, and such abnormal expression was reversed by BAM15 (Figs. 1G and S3C). Additionally, BAM15 corrected spindle migration defects and reduced excessive actin levels in obese oocytes (Figs. 1H, 1I, S3D and S3E). Actin regulatory DEGs were closely linked to core pathways (Fig. S3F). Obesity elevated cortical and cytoplasmic actin regulators as well as ROCK and p-Cofilin expression, while BAM15 restored their homeostasis (Figs. 1J, S3G and S3H). These results suggested that BAM15 rescued obesity-induced cytoskeletal defects in oocytes. We further explored the protective effects of BAM15 on mitochondrial function in obese oocytes. BAM15 markedly ameliorated the mitochondrial abnormalities of diffuse or aggregated distribution, reduced fluorescence intensity and lowered membrane potential in obese oocytes (Figs. 1K, 1L, S4A and S4B). The mitochondrial functional DEG *Tnfsf10* interacted with the fission protein DRP1 (Fig. S4C). Obesity inhibited pS616-DRP1 expression and disrupted its spindle localization while abnormally activating the mitophagy protein Parkin, whereas BAM15 restored pS616-DRP1 expression and localization, ameliorated aberrant Parkin activation, and rescued mitochondrial dynamics and mitophagy homeostasis (Figs. 1M and S4D–F). Moreover, BAM15 reduced ROS accumulation and oxidative stress, downregulated γ-H2A.X levels, and alleviated DNA damage in obese oocytes (Figs. 1N–P and S4G). These results demonstrated that BAM15 ameliorated mitochondrial dysfunction, oxidative stress and DNA damage in obese oocytes.

Next, we investigated how BAM15 improves obese oocyte quality. KEGG enrichment identified the PPAR signaling pathway as the core differential pathway (Figs. 1D and S2F). GSEA confirmed significant downregulation of this pathway in obesity, which was reversed and upregulated by BAM15 intervention (Fig. 2A). We focused on PPARγ, a center regulator of lipid metabolism and energy homeostasis in the PPAR family. Molecular docking confirmed that BAM15 could stably bind to the ligand-binding domain of PPARγ (Fig. 2B). Immunofluorescence and protein assays validated reduced nuclear localization and expression of PPARγ in obese oocytes, both rescued by BAM15 (Fig. 2C and 2D). The PPARγ upstream regulator PEX3 was downregulated in obesity and restored by BAM15 (Figs. 2D and S5A). Consistently, the mRNA levels of *Pparγ* and its downstream target genes *Nrf2* and *Pgc1α* were inhibited in obesity and recovered following BAM15 treatment (Fig. 2E; Table S1). To verify the regulation of PPARγ by BAM15, we performed experiments using its agonist and antagonist. Low-concentration rosiglitazone had no significant effect on oocyte GVBD but markedly increased the PB1 extrusion rate. Different concentrations of GW9662 inhibited PB1 extrusion or GVBD to mimic the maturation defects of obese oocytes, and BAM15 effectively reversed the maturation abnormalities caused by the antagonist (Figs. 2F–H and S5B–D). Further mechanism assays showed rosiglitazone-induced PPARγ activation alleviated mitochondrial aggregation, restored mitochondrial membrane potential and reduced ROS accumulation in obese oocytes (Figs. 2I, 2K and S5E). GW9662 treatment recapitulated obesity-related mitochondrial damage in normal oocytes, which was rescued by combined BAM15 treatment (Figs. 2J, 2L and S5F). These results showed that BAM15 activated the PPARγ signaling pathway to restore mitochondrial function, which enhances the quality of obese oocytes.

**Figure 2.**
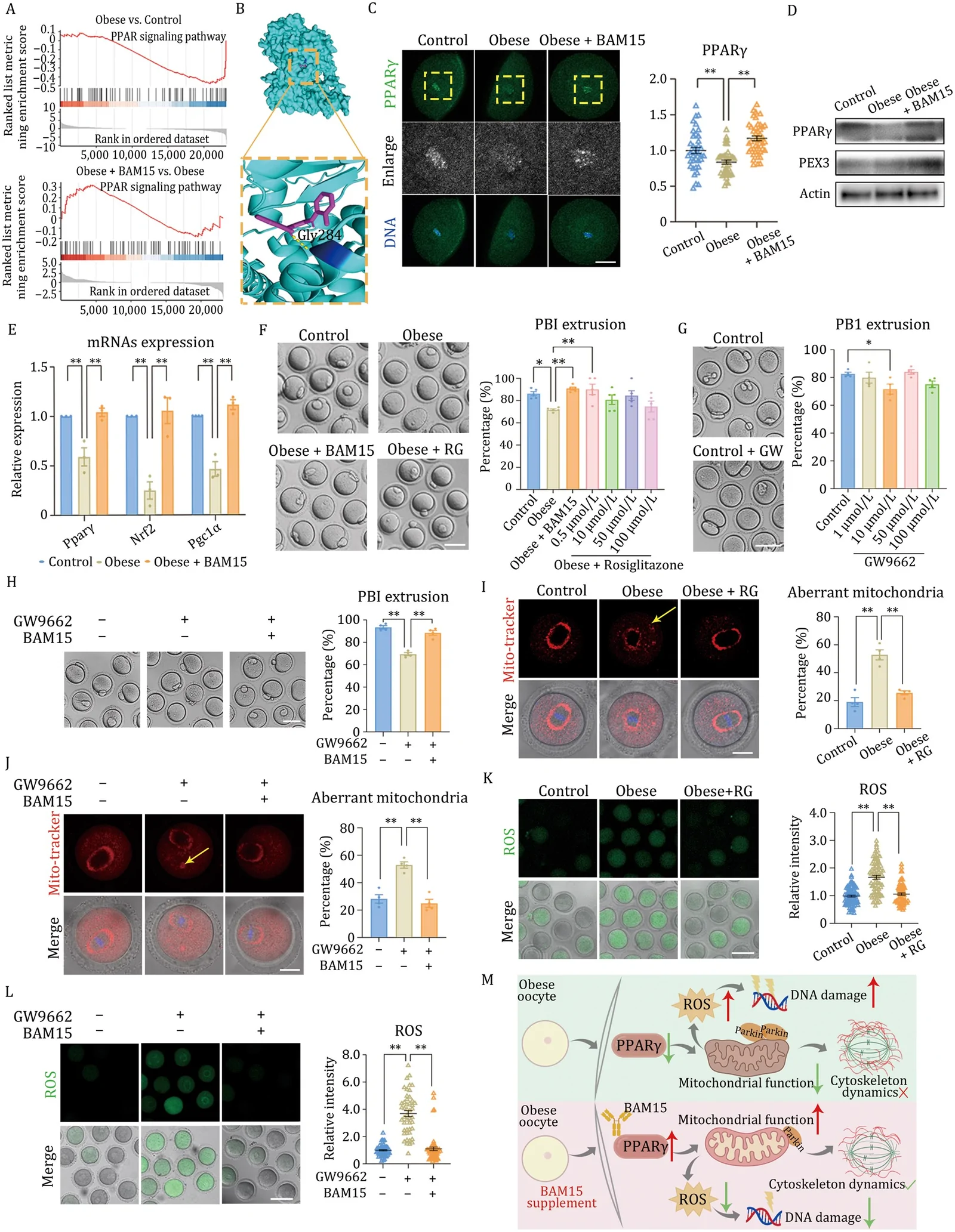
**BAM15 improves mitochondrial function via the PPARγ signaling pathway in obese oocytes**. (A) Gene Set Enrichment Analysis (GSEA) based on the normalized gene expression levels of the PPAR signaling pathway in oocytes from control, obese, and BAM15-treated groups. (B) Computational molecular docking analysis of BAM15 bound to the PPARγ ligand-binding domain. (C) Representative images and relative fluorescence intensity of PPARγ in oocytes from control (*n *= 38), obese (*n *= 42), and obese + BAM15 (*n *= 43) groups. Bar = 20 μm. (D) Representative western blot bands of PPARγ and PEX3 in MI stage oocytes from control, obese, and obese + BAM15 groups. (E) The mRNA level of genes *Pparγ*, *Nrf2*, and *Pgc1α* in oocytes from control, obese, and obese + BAM15 groups. (F) Representative images of PB1 extrusion in control, obese, obese + BAM15 oocytes, and obese + 0.5 μmol/L rosiglitazone oocytes, with quantitative analysis PB1 extrusion rates across control (*n *= 126), obese (*n *= 141), obese + BAM15 (*n *= 132), and obese oocytes supplemented with 0.5 μmol/L (*n *= 119), 10 μmol/L (*n *= 130), 50 μmol/L (*n *= 124), or 100 μmol/L (*n *= 122) rosiglitazone during IVM. Bar = 80 μm. (G) Representative images of PB1 extrusion in Control and 10 μmol/L GW9662 oocytes, with quantitative analysis of PB1 extrusion rates across control (*n *= 135), 1 μmol/L (*n* = 138), 10 μmol/L (*n *= 134), 50 μmol/L (*n *= 139), and 100 μmol/L (*n *= 137) GW9662 during IVM. GW, GW9662. Bar = 80 μm. (H) Representative images and quantitative analysis of PB1 extrusion in control (*n *= 106), GW9662 (*n *= 106), and GW9662 + BAM15 (*n *= 108) oocytes during *in vitro* maturation. Bar = 80 μm. (I) Representative images and percentage of abnormal mitochondria (indicated by yellow arrows) in oocytes from control (*n *= 73), obese (*n *= 70) and obese + rosiglitazone (*n *= 62) groups. Blue, DNA. Bar = 20 μm. (J) Representative images and percentage of abnormal mitochondria (indicated by arrows) in oocytes from control (*n *= 58), GW9662 (*n *= 61), and GW9662 + BAM15 (*n *= 57) groups. Blue, DNA. Bar = 20 μm. (K) Representative images and relative fluorescence intensity of ROS in oocytes from control (*n *= 79), obese (*n *= 70) and obese + rosiglitazone (*n *= 72) groups. Bar = 80 μm. (L) Representative images and relative fluorescence intensity of ROS in oocytes from control (*n *= 58), GW9662 (*n *= 47), and GW9662 + BAM15 (*n *= 57) groups. Bar = 80 μm. (M) Diagram depicting BAM15-activated PPARγ-mediated mitochondrial restoration for ameliorating obese oocyte quality. Data are presented as mean ± SEM of three biological repeats. *, *P *< 0.05. **, *P *< 0.01.

In the present study, we report for the first time the protective effects and underlying molecular mechanisms of BAM15 on reproductive function in obese mice. We found that BAM15 targeted PPARγ to restore mitochondrial function, alleviate oxidative stress and maintain cytoskeletal stability, which improved the maturation quality of obese oocytes and enhanced embryonic developmental potential (Fig. 2N).

Obesity impairs mammalian oocyte quality, reducing the meiotic maturation rate and embryonic developmental potential (Han et al., 2017; Hou et al., 2016). As a novel mitochondrial uncoupler, BAM15 outperforms traditional uncouplers with higher mitochondrial targeting efficiency and fewer off-target effects (Kenwood et al., 2014). Here, *in vitro* supplementation with BAM15 significantly promoted oocyte maturation and improved embryonic development. To explore the underlying mechanism, we performed transcriptome sequencing, which revealed that DEGs were mainly enriched in cytoskeleton-related cellular components. While obesity directly destroys the structural integrity of the oocyte spindle (Hou et al., 2016). This study confirmed that BAM15 repaired cytoskeletal disorders in obese oocytes. This is consistent with its functions in somatic cells, as BAM15 protects microtubules against cold injury in rat retinal ganglion cells and refrigerated mouse kidneys and stabilizes actin in obese mouse skeletal muscle (Dantas et al., 2022; Ou et al., 2018). Mitochondria supply energy for oocyte vital activities (van der Reest et al., 2021), whereas obesity induces mitochondrial distribution disorder and dysfunction in oocytes (Han et al., 2017; Hou et al., 2016; Wang et al., 2022). Similar to NMN, which enhances mitochondrial function in obese oocytes (Wang et al., 2022), BAM15 alleviated obesity-related mitochondrial dysfunction in our study. In obese mice, DRP1-mediated mitochondrial dynamics imbalance suppresses antioxidant capacity and elevates ROS levels (Wu et al., 2025). We further demonstrated that BAM15 relieved oxidative stress and reduced DNA damage by activating Parkin-dependent mitophagy to maintain redox homeostasis, consistent with the reported mechanism whereby BAM15 induces mitophagy via the PINK1 ubiquitination pathway in skeletal muscle (Dantas et al., 2022). Given the regulatory role of mitochondria in transcription and mRNA levels (Cheng et al., 2022), mediating key processes including spindle assembly and redox homeostasis, BAM15 remodels the oocyte mRNA profiles by improving mitochondrial function, which likely accounts for the diverse gene expression patterns linked to multiple cellular events in our analysis.

KEGG analysis revealed significant enrichment of the PPAR signaling pathway as the target pathway of BAM15. PPARγ is a core regulator of lipid metabolism and energy homeostasis, and neuronal PPARγ knockout impairs fertility in obese mice (Fernandez et al., 2017). We showed that PPARγ activity is critical for oocyte maturation in obesity, and our findings revealed that BAM15 supplementation markedly upregulates PPARγ expression in obese oocytes. Given the close correlation between obesity and mitochondrial dysfunction in oocytes, we speculate that PPARγ inactivation is a vital mechanism underlying mitochondrial homeostasis imbalance and subsequent oocyte developmental defects, and that BAM15 can reverse these impairments by activating this pathway. Previous studies have verified that PPARγ is pivotal for maintaining mitochondrial function, and PPARγ knockdown induces mitochondrial structural and functional disorders as well as aggravated oxidative stress in pulmonary vascular cells (Yeligar et al., 2018). Meanwhile, tocopherols can promote mitochondrial biogenesis by activating PPARγ in adipose tissue of obese rodents, further supporting the conservation of this regulatory pathway (Cremonini et al., 2024). Collectively, BAM15 ameliorated obesity-induced oocyte maturation defects by activating PPARγ to restore mitochondrial function.

In conclusion, our data suggested that BAM15 activated PPARγ pathway to enhance mitochondrial function, alleviating oxidative stress-induced DNA damage, which protect cytoskeletal for oocyte quality in obese mice.

## Supplementary Material

pwag035_Supplementary_Data

## Footnotes

This work was supported by the National Natural Science Foundation of China (32571001), the Fundamental Research Funds for the Central Universities of China (KJJQ2026001, RENCAI2025035), and National Key Research and Development Program of China (2021YFC2700100). The diagram was created with BioGDP.com.

This study has been approved by the committee of Animal Ethics and Farewell in Nanjing Agricultural University (NJAU.No20231206184). All institutional and national guidelines for the care and use of laboratory animals were followed.

The data that support the findings of this study are available from the corresponding author upon reasonable request. The authors declare no conflict of interest.

S.C.S. and R.J.M. conceived the study. R.J.M. performed the experiments. R.J.M. analyzed the data. H.L.Z., X.W., P.S.L., K.H.Z., Y.X.H., Z.J.W., and Y.W. contributed to materials and agents. R.J.M. and S.C.S. wrote the manuscript.

The data that support the findings of this study are available from the corresponding author upon reasonable request.
