# Supplementary material for: BAM15 improves oocyte quality against obesity via PPARγ-dependent mitochondrial function
Source: Protein Cell. 2026 May 24;17(11):1078–83. doi: 10.1093/procel/pwag035 (PMC13721119; doi:10.1093/procel/pwag035)
Supplement: Supplementary file 1 [file pwag035_supplementary_data.pdf]

Supplementary Materials for

BAM15 improves oocyte quality against obesity via PPARγ-dependent mitochondrial function

Rui-Jie Ma[1], Hao-Lin Zhang[1], Xuan Wu[1], Ping-Shuang Lu[1], Kun-Huan Zhang[1], Yu-Xuan Hou[1], Zi-Jian Wu[1], Yue Wang[1], Shao-Chen Sun[1, 2*]

[1]College of Animal Science and Technology, Nanjing Agricultural University, Nanjing 210095, China.

[2]Key Laboratory of Research on Clinical Molecular Diagnosis for High Incidence Diseases in Western Guangxi of Guangxi Higher Education Institutions, Affiliated Hospital of Youjiang Medical University for Nationalities, Baise 533000, China.

***Correspondence to:** Shao-Chen Sun, College of Animal Science and Technology, Nanjing Agricultural University, Nanjing, China. Tel/Fax: +86-25-84399092. E-mail: sunsc@njau.edu.cn

Materials and Methods

Ethics statement and animals

All procedures were approved by the Animal Ethics Committee of Nanjing Agricultural University (NJAU.No20231206184) and strictly performed in accordance with the guidelines of its Animal Research Committee (License No. XYXK-Su-2017-0007). Female Institute of Cancer Research (ICR) female mice (6 weeks old) were purchased from Henan Skobes Biotechnology Co., Ltd. (license no. SCXK-Yu-2020-0005), a government-licensed provider. ICR male mice (11–12 weeks old) were obtained from Jiangsu Qinglongshan Biotechnology Co., Ltd. (license no. SCXK-Su-2024-0001). All mice were maintained in a standard light/dark cycle for >1 week acclimation. Mice were fed a 60% kcal HFD (#SN673477070876741, Qinglongshan Biotech) for 12 weeks, with control groups receiving standard chow (10% kcal fat). Diet-induced obese models were defined at 12 weeks as HFD-fed mice showing > 20% higher body weight than controls ($P < 0.05$). All animals were housed under standard conditions with ad libitum access to food and water throughout the study.

Antibodies and chemicals

Anti-acetylated tubulin antibody (T7451), an anti-α-tubulin-FITC antibody (F2168) and Hoechst 33342 (B2261) were purchased from Sigma-Aldrich. Anti-Formin2 (11259-1-AP) antibody and anti-Parkin antibody (14060-1-AP) were purchased from Proteintech. Anti-γ-H2A.X antibody (ab81299), anti-Parkin antibody (ab77924), anti-ROCK antibody (ab45171), anti-ARP2 antibody (ab128934), anti-N-WASP antibody (ab126626) and anti-γ-tubulin antibody (ab179503) were purchased

from Abcam. Anti-phospho-DRP1 (Ser616) antibody (3455), anti-phospho-Cofilin (Ser3) antibody (3313), anti-PPARγ antibody (2443) and anti-β-Actin antibody (3700) were purchased from Cell Signaling Technology. FITC- and TRITC-conjugated goat anti-rabbit IgG (H + L) secondary antibodies were obtained from Zhongshan Golden Bridge Biotechnology. Anti-HDAC6 antibody (a11259) was obtained from ABclonal Technology. Anti-Peroxin 3 antibody (ER64562) was commercially acquired from HUABIO. FITC-conjugated Lens culinaris agglutinin (L32475) was obtained from Invitrogen. Mito-tracker (M7512) and Hoechst 33342 for dead cells (H3570) were from Life Technologies. TMRE-Mitochondrial Membrane Potential Assay Kit (C2001S), Hoechst 33342 for live cells (C1027), and ROS Assay Kit (DCFH-DA, S0033) were obtained from Beyotime Biotechnology. HRP-conjugated goat antirabbit/mouse antibodies (CW0103/CW0102) were obtained from CWBIO. BAM15 (HY-100798) and Rosiglitazone (HY-17386) were purchased from MedChemExpress. All other unstated chemicals were from Sigma.

Oocyte collection and in vitro maturation

After cervical dislocation, GV oocytes were isolated from mouse ovaries, sequentially washed in M2 three times and M16 media, and then cultured in mineral oil-overlaid M16 medium at 37°C with 5% CO_2. Oocyte maturation progression showed MI stage at 8 h and MII stage at 12 h of incubation.

BAM15, Rosiglitazone and GW9662 treatment

The stock solutions of BAM15, rosiglitazone and GW9662 were 10 mM (1 mL in DMSO). All stock solutions were aliquoted into single-use portions, stored at −80 °C

in light-proof cryovials to prevent degradation. Working solutions were freshly prepared by diluting the stock solutions with M16 medium immediately before use. The final concentration of DMSO was kept below 0.1% in all experiments to avoid cytotoxicity. During *in vitro* maturation (IVM) of GV stage oocytes to MI or MII stages, BAM15, rosiglitazone, or GW9662 were individually supplemented into M16 culture medium as required by experimental design. For oocyte fertilization and subsequent embryonic development, BAM15 was added to the following sequential media: TYH medium (for sperm capacitation and fertilization), HTF medium (for zygote culture), KSOM medium (for blastocyst formation).

***In vitro* fertilization and early embryonic development**

Female mice were primed with 10 IU PMSG for 48 h, followed by 10 IU hCG injection for superovulation. Sperm were collected from males 13-16 h after hCG administration and capacitated in TYH medium for 1 h. Meanwhile, cumulus-oocytes complexes (COCs) were isolated from oviductal ampullae and placed in pre-equilibrated HTF medium. Gametes were co-incubated in HTF medium at 37 °C with 5% CO_2 for 6 h. After fertilization confirmation, zygotes were washed three times in fresh KSOM medium and continuously cultured in a 5% CO_2 incubator at 37 °C until blastocyst formation.

Immunofluorescence staining and confocal microscopy

Denuded oocytes were sequentially processed as follows: fixed in 4% paraformaldehyde (PFA) for 30 min， permeabilized with 0.5% Triton X-100 in PBS for 20 min and blocked with 1% bovine serum albumin (BSA) in PBS for 1 h at room

temperature. After blocking, samples were incubated with primary antibodies at 4°C overnight. The primary antibodies included: FITC-conjugated anti-α-tubulin (1:400), anti-γ-tubulin (1:500), anti-acetylated tubulin (1:400), anti-γ-H2A.X (1:250), anti-Parkin (1:200), anti-phospho-DRP1 (Ser616) (1:100), anti-PPARγ (1:100). After three washes with washing buffer, samples were incubated with corresponding secondary antibodies for 1 h at room temperature: Alexa Fluor 488/TRITC-conjugated goat anti-rabbit IgG (1:200) or Alexa Fluor 488/TRITC-conjugated goat anti-mouse IgG (1:200). For F-actin staining, oocytes were incubated with rhodamine-phalloidin (1:200) for 1 h at room temperature. Following three additional washes, nuclei were counterstained with Hoechst 33342 (1:10,000) for 10 min at room temperature. Finally, samples were mounted on glass slides and imaged using a Zeiss LSM 800 META confocal laser scanning microscope (Jena, Germany).

Live-cell fluorescence staining was performed to examine the distribution of mitochondria, mitochondrial membrane potential and ROS levels. For the detection of mitochondrial distribution, mitochondrial membrane potential using TMRE, and ROS levels, MI-stage oocytes were washed three times in M2 medium and then incubated with the following probes diluted in M16 medium at 4 °C for 30 min including Mito-Tracker (1:800), TMRE Assay (1:200) and ROS Assay (1:600). After staining, the oocytes were further incubated with Hoechst 33342 diluted in M16 medium at 37 °C with 5% CO_2 for 30 min and immediately imaged under a confocal microscope.

For cortical granule, MII-stage oocytes were treated with pronase at 37°C for 5 min to remove the zona pellucida, followed by fixation in 4% PFA for 30 min. After

fixation, the oocytes were washed three times (5 min each) in PBS containing 0.3% BSA and 100 mM glycine, then permeabilized with 0.1% Triton X-100 for 5 min. Subsequently, the samples were washed twice in PBS (5 min each) and incubated with LCA-FITC staining solution (1:100 dilution in PBS) for 1 h at room temperature. Following LCA-FITC staining, the oocytes were washed three times (5 min each) in PBS containing 0.3% BSA and 0.01% Triton X-100. Finally, DNA was stained with Hoechst 33342, and fluorescence signals were examined as previously described.

Fluorescence intensity analysis

Image J software (National Institutes of Health, Bethesda, MD, USA) was used for fluorescence intensity measurement. For each experiment, samples from the control, obese, and obese + BAM15 groups were scanned under identical parameters. Target regions were selected as regions of interest (ROI) for analysis, and fluorescence intensity was measured using ZEN lite 2012 software and ImageJ software (National Institutes of Health, Bethesda, MD, USA).

RNA extraction and quantitative real time

A total of 50 MI-stage oocytes were collected. Total RNA was extracted using the Dynabeads® mRNA DIRECT™ Micro Kit (Invitrogen Dynal AS, Norway), and cDNA was synthesized by reverse transcription with PrimeScript™ RT Master Mix (Takara). The qPCR amplification protocol was set as follows: initial denaturation at 95℃ for 30 sec, followed by 40 cycles of amplification (95℃ for 5 sec, 60℃ for 30 sec), and melt curve analysis (95℃ for 5 sec, 60℃ for 60 sec, 95 ℃ for 1 sec). The final products were held at 4℃. Relative gene expression levels were calculated using the $2^{-\Delta\Delta Ct}$

method with *glyceraldehyde-3-phosphate dehydrogenase (*Gapdh*)* as the internal reference gene. Primer sequences used in this study are listed in Table S1.

Western blot analysis

A total of 180 MI-stage oocytes were collected and lysed using NuPAGE LDS Sample Buffer. The lysates were then heated at 100°C for 10 min in a metal bath to prepare western blot samples. Protein separation was performed by electrophoresis on 10% SurePAGE gels (M00665, GenScript Biotech Co.) at 160 V for 1 h. Subsequently, proteins were transferred onto polyvinylidene fluoride (PVDF) membranes (Millipore, Billerica, MA) using the eBlotTM L1 transfer system (L00686C, GenScript Biotech Co.). Following protein transfer, the membranes were blocked at room temperature for 15 min using QuickBlock™ Blocking Buffer (P0252, Beyotime Biotechnology). The membranes were then incubated overnight at 4 °C with the corresponding primary antibodies at optimized dilutions. The next day, membranes were washed three times with TBST and incubated for 1 h at room temperature with horseradish peroxidase (HRP)-conjugated secondary antibodies (anti-mouse or anti-rabbit, 1:2000). After three additional TBST washes, protein bands were visualized using the ECL Plus Western blot Detection System (Tanon-3900) and quantified with Image J software (National Institutes of Health). For re-probing, the membranes were stripped of primary and secondary antibodies using Restore™ PLUS western blot Stripping Buffer (46430, Thermo Fisher Scientific).

Single-cell RNA sequencing and analysis

Firstly, the isolated oocytes were lysed by adding them to the lysis buffer. Subsequently, reverse transcription PCR (RT-PCR) amplification was performed by adding MMLV reverse transcriptase, free dNTPs, oligo (dT) VN primer, betaine, $MgCl_2$, and template-switching oligonucleotide (TSO) to the lysate to generate double-stranded cDNA. The cDNA was then mechanically fragmented using standard methods, and small fragments were selected before ligation with sequencing adapters. The library was constructed through RT-PCR amplification and magnetic bead purification. Quality-approved libraries were sequenced on the Illumina platform, sequencing performed by Annoroad Gene Technology. The web-based analytical platforms Microbioinformatics (https://www.bioinformatics.com.cn/) and Chiplot (https://www.chiplot.online/) were utilized in this study for bioinformatics analyses, including statistical analysis of biological data, Gene Ontology (GO) enrichment analysis, Kyoto Encyclopedia of Genes and Genomes (KEGG) pathway analysis, and data visualization.

Statistical analysis

All experiments were performed with at least three biological replicates. Statistical analysis was conducted using one-way ANOVA via GraphPad Prism 9.5.0 software (GraphPad, San Diego, CA). Data were presented as mean ± SEM, and the sample numbers of mice or oocytes (n) are shown in parentheses. Statistical significance was defined as $P < 0.05$.

Supplementary Figure and Legends

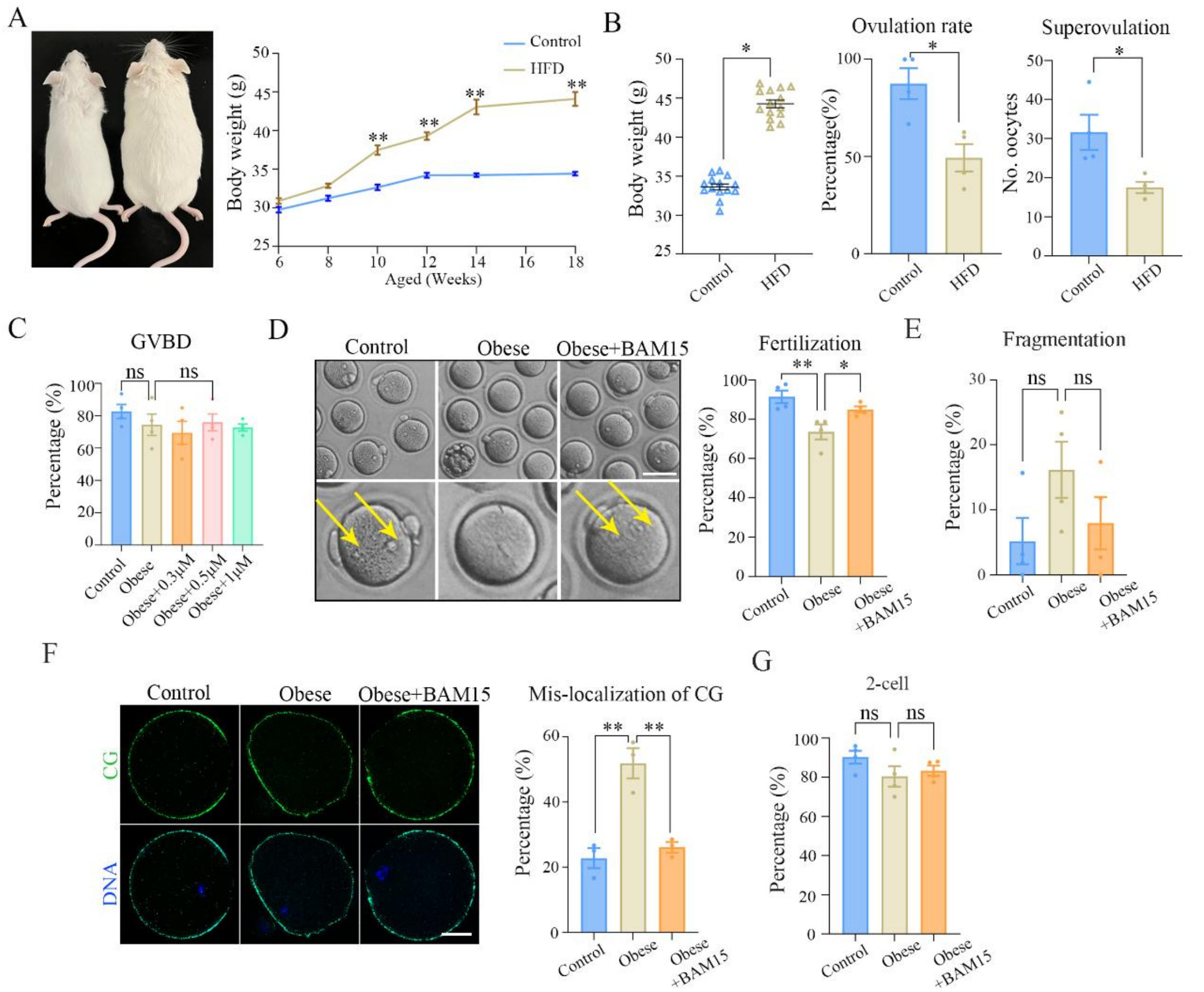

Fig. S1. Establishment of the obese mouse model and rescue of oocyte quality by BAM15 treatment. (A) The typical images of mice with standard chow and high fat diet; body weight curves of control and HFD-fed mice during the 12-week experimental period. (B) Comparison of body weight, ovulation rate and oocyte yield between obese and control mice (body weight, control n=14, obese n=14; ovulation rate, control n=32, obese n=31; oocyte yield, control n=11, obese n=15). (C) The rates of GVBD were calculated after 4 h of in vitro maturation (IVM) in the control (n=127), obese (n=145), and obese groups treated with 0.3, 0.5 and 1.0 μM BAM15 (n=130, 130, 132). (D) Representative images and quantitative analysis of pronuclear formation (indicated by yellow arrows) after in vitro fertilization (IVF) for oocytes from the control (n = 192), obese (n = 174), and obese + BAM15 (n = 170) groups. Bar = 80 μm. (E) Quantitative

analysis of the fragmentation rate in MII oocytes among the control (n = 203), obese (n = 201), and obese + BAM15 (n = 187) groups following IVF. The fragmentation rate was determined 6h post-fertilization of MII oocytes. (F) Representative images and percentage of abnormal cortical granule (CG) in oocytes from control (n = 43), obese (n = 60), and obese + BAM15 (n = 42) groups. Bar = 20 μm. (G) Quantitative analysis of 2-cell formation rates at 24 h after IVF for the control (n = 175), obese (n = 126), and obese + BAM15 (n = 144) groups. The 2-cell formation rates were calculated based on fertilized zygotes. Data are presented as mean ± SEM of three biological repeats. ns, $P > 0.05$. *, $P < 0.05$. **, $P < 0.01$.

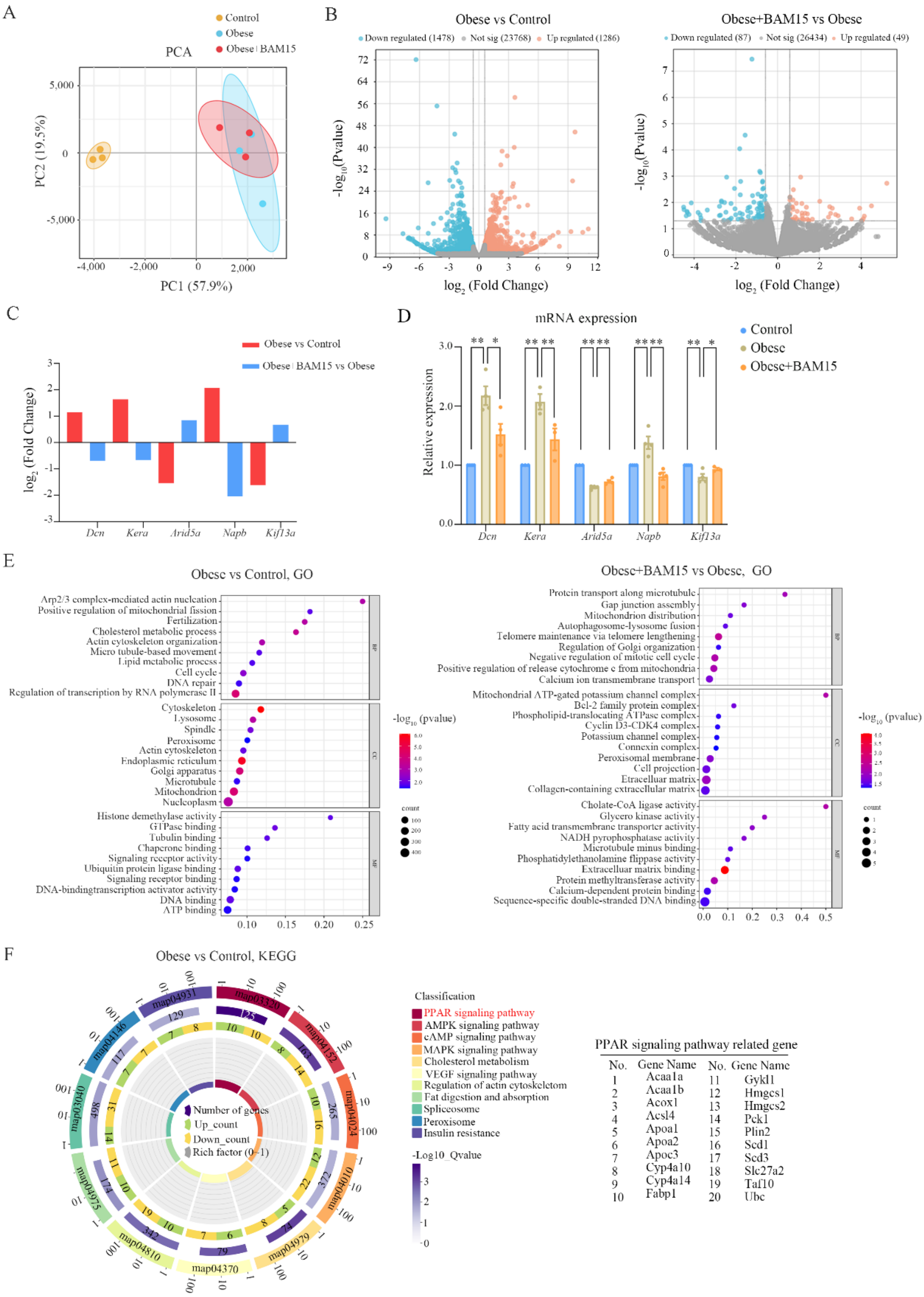

Fig. S2. Effects of BAM15 supplementation on transcriptional profiles in oocytes from obese mice. (A) PCA of oocyte samples from control, obese, and obese + BAM15 groups. (B) Volcano plots displaying transcriptomic changes between: control vs. obese

oocytes, and obese + BAM15 vs. obese oocytes with downregulated (blue) and upregulated (red) differentially expressed genes (DEGs) indicated. (C) RNA-seq results of selected DEGs in oocytes from control, obese and obese + BAM15 groups. (D) Relative mRNA expression of randomly selected genes including *Dcn*, *Kear*, *Arid5a*, *Napb* and *Kif13a* in oocytes from the control, obese, and obese + BAM15 groups. (E) GO enrichment analysis of DEGs between: control vs. obese oocytes, and obese + BAM15 vs. obese oocytes. (F) KEGG enrichment analysis of DEGs between obese and control oocytes, with a list of PPAR pathway-related genes.

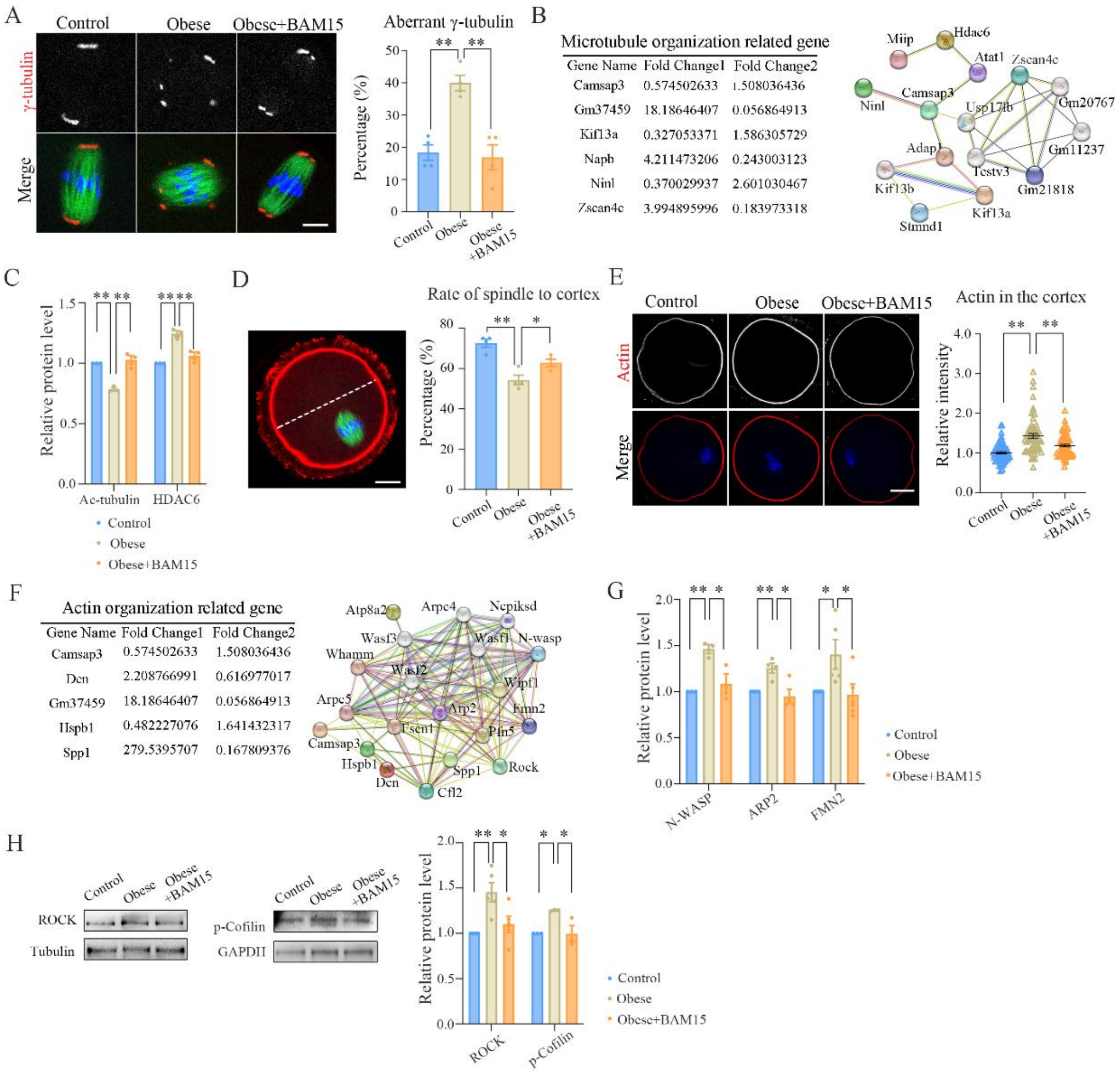

Fig. S3. BAM15 improves cytoskeletal dynamics in obese oocytes. (A) Representative images and percentage of abnormal γ-tubulin in oocytes from control (n = 59), obese (n = 60), and obese + BAM15 (n = 54) groups. Blue, DNA. Bar = 10 μm. (B) DEGs related to microtubule function were identified across control, obese, and obese + BAM15 groups; protein-protein interaction (PPI) network analysis of HDAC6 and microtubule-related DEGs. (C) Band intensity analysis of HDAC6 and ac-tubulin in MI stage oocytes from the control, obese, and obese + BAM15 groups. (D) Representative images and percentage of spindle migration toward the cortex in oocytes from control (n = 69), obese (n = 70) and obese + BAM15 (n = 70) groups. Green, α-tubulin. Red, actin. Blue, DNA. Bar = 20 μm. (E) Representative images and relative fluorescence intensity of cortical actin filaments in oocytes from control (n = 69), obese (n = 60), and obese + BAM15 (n = 67) groups. Blue, DNA. Bar = 20 μm. (F) DEGs related to actin function were identified across control, obese, and obese + BAM15 groups; PPI network analysis of actin-associated proteins and actin-related DEGs. (G) Band intensity analysis of N-WASP, ARP2 and FMN2 in MI stage oocytes from the control, obese, and obese + BAM15 groups. (H) Representative western blot images and band intensity analysis of ROCK and p-cofilin in MI stage oocytes of control, obese, and obese + BAM15 groups. Data are presented as mean ± SEM of three biological repeats. *, $P < 0.05$. **, $P < 0.01$.

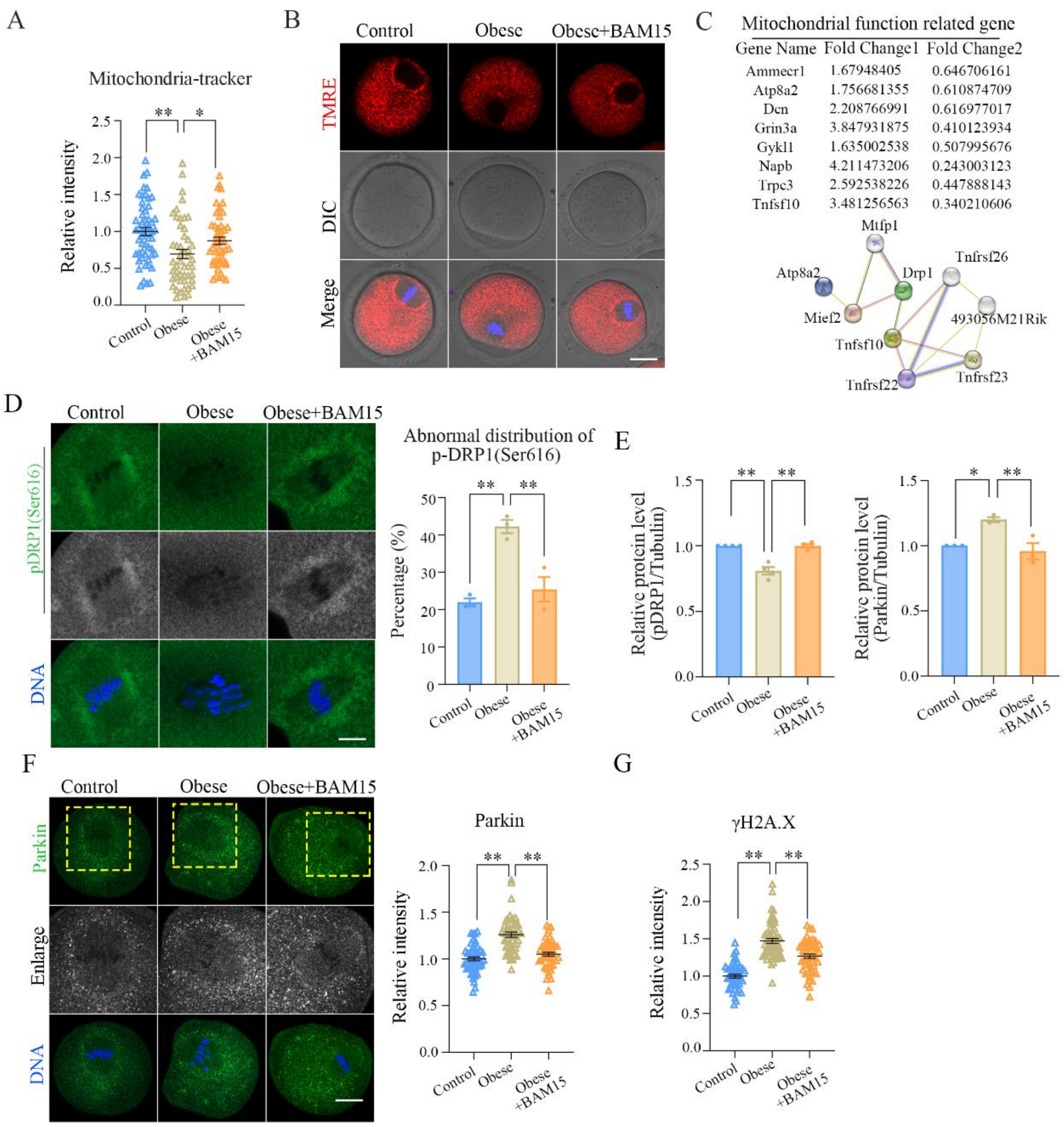

Fig. S4. BAM15 ameliorates mitochondrial dysfunction in obese oocytes. (A) Relative fluorescence intensity of mitochondria in oocytes from control (n = 55), obese (n = 49) and obese + BAM15 (n = 52) groups. (B) Representative images of TMRE in oocytes from control, obese and obese + BAM15 groups. Blue, DNA. Bar = 20 μm. (C) DEGs related to mitochondrial function were identified across control, obese, and obese + BAM15 groups; PPI network analysis of mitochondrial fission protein DRP1 and mitochondria-related DEGs. (D) Representative images and percentage of abnormal pS616-DRP1 in oocytes from control (n = 68), obese (n = 52) and obese + BAM15 (n

= 55) groups. Bar = 10 μm. (E) Band intensity analysis of pS616-DRP1 and Parkin in MI stage oocytes from the control, obese, and obese + BAM15 groups. (F) Representative images and relative fluorescence intensity of Parkin in oocytes from control (n = 51), obese (n = 49) and obese + BAM15 (n = 49) groups. Bar = 20 μm. (G) Relative fluorescence intensity of γ-H2A.X in oocytes from control (n = 47), obese (n = 57) and obese + BAM15 (n = 52) groups. Data are presented as mean ± SEM of three biological repeats. *, $P < 0.05$. **, $P < 0.01$.

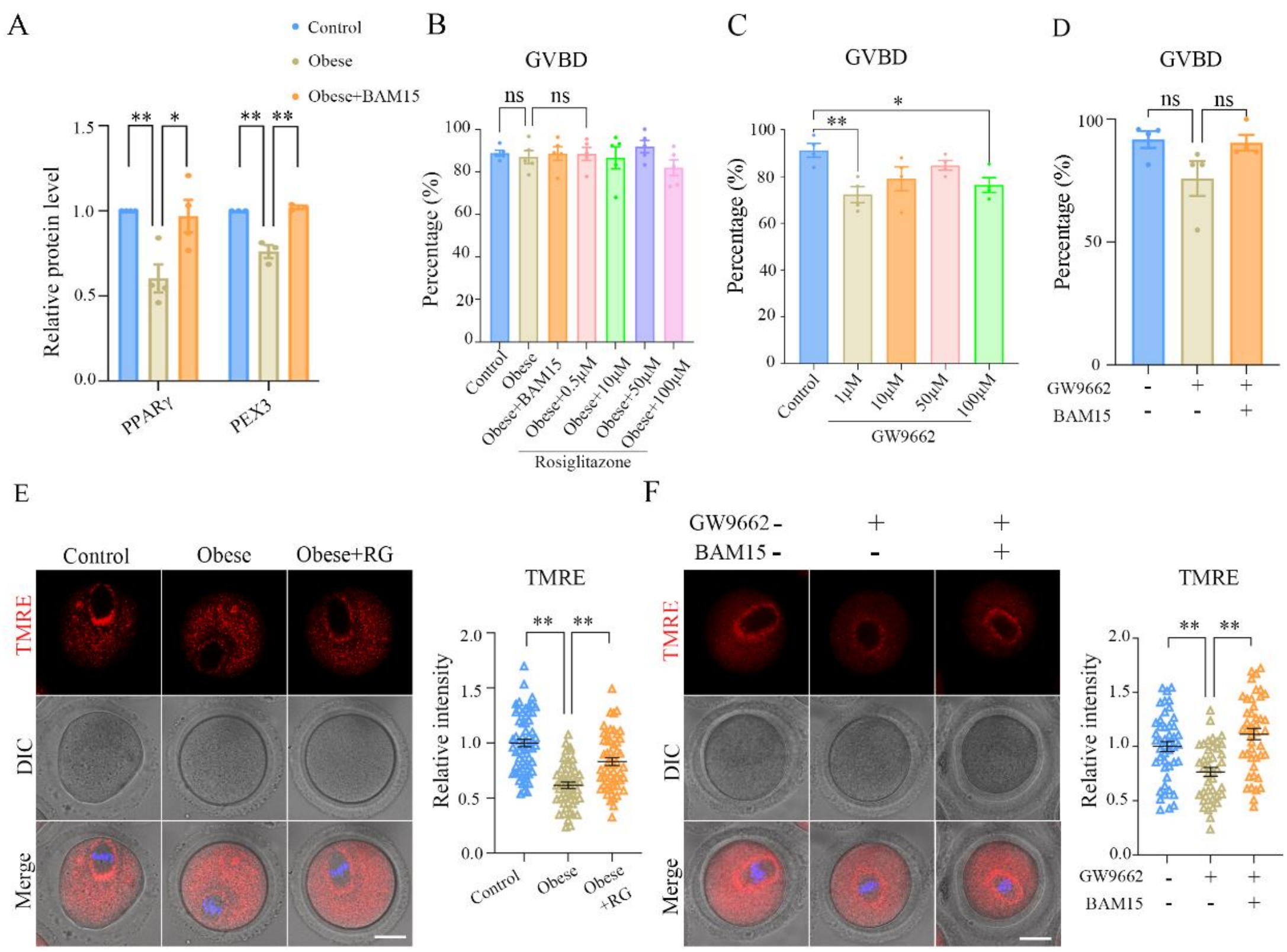

Fig. S5. BAM15 ameliorates mitochondrial dysfunction by regulating the PPARγ signaling pathway in obese oocytes. (A) Band intensity analysis of PPARγ and PEX3 in MI stage oocytes from the control, obese, and obese + BAM15 groups. (B) Quantitative analysis GVBD rates across control (n = 126), obese (n = 141), obese +

BAM15 (n = 132), and obese oocytes supplemented with 0.5 μM (n = 119), 10 μM (n = 130), 50 μM (n = 124), or 100 μM (n = 122) rosiglitazone during IVM. (C) Quantitative analysis of GVBD in oocytes during IVM under different GW9662 concentrations: control (n = 135), 1 μM (n = 138), 10 μM (n = 134), 50 μM (n = 139), and 100 μM (n = 137). (D) Quantitative analysis of GVBD in control (n = 106), GW9662 (n = 106), and GW9662 + BAM15 (n = 108) oocytes during IVM. (E) Representative images and relative fluorescence intensity of TMRE in oocytes from control (n = 60), obese (n = 50) and obese + rosiglitazone (n = 53) groups. Blue, DNA. Bar = 20 μm. (F) Representative images and relative fluorescence intensity of TMRE in oocytes from control (n = 46), GW9662 (n = 41) and GW9662 + BAM15 (n = 44) groups. Blue, DNA. Bar = 20 μm. Data are presented as mean ± SEM of three biological repeats. ns, $P > 0.05$. *, $P < 0.05$. **, $P < 0.01$.

Table S1. The specific primers used in the study

Gene	Forward primer (5'-3')	Reverse primer (5'-3')
Gapdh	AGGTCGGTGTGAACGGATTTG	TGTAGACCATGTAGTTGAGGTCA
Dcn	ACCTCTCGTGAAGTTGGAAAGG	CCCAGAGTTTTTCAGTGGGTTG
Kera	ATGGCGAAGAATGCCCTGAG	AGCCACCTTAGGAATCACATTG
Kif13a	GTAGCAGCGACCAACATGAAT	CCAGTCTTCGACACTCTCTCG
Arid5a	CCACTCACGGGTGCTAGTAG	GGGGACTGTCTTGAATGTCCT
Napb	GCCAAGCACCATATCACCATT	GCAGAGCTGTTAGATTCCTCTCC

Pparγ	TCCGAAGAACCATCCGATTGA	AACAGCTTCTCCTTCTCGGC
Nrf2	TAGATGACCATGAGTCGCTTGC	GCCAAACTTGCTCCATGTCC
Pgc1a	TATGGAGTGACATAGAGTGTGCT	GTCGCTACACCACTTCAATCC
